# Development of bioabsorbable polylactide membrane with controllable hydrophilicity for adjustment of cell behaviours

**DOI:** 10.1098/rsos.170868

**Published:** 2018-01-17

**Authors:** Yang Yang, Xiaofeng Qiu, Yi Sun, Yifeng Wang, Jine Wang, Yulin Li, Changsheng Liu

**Affiliations:** The Key Laboratory for Ultrafine Materials of Ministry of Education, State Key Laboratory of Bioreactor Engineering, Engineering Research Center for Biomedical Materials of Ministry of Education, East China University of Science and Technology, Shanghai 200237, People's Republic of China

**Keywords:** ring opening polymerization, poly(d,l-lactide) membrane, amphiphilic poly(ethylene glycol)-b-poly(d,l-lactide), surface hydrophilicity, biodegradation, cell behaviours

## Abstract

Cell functions can be mediated through their interactions with the microenvironments, which highly depend on the surface state of the substrate. However, how to finely adjust the surface of biomaterials is still very challenging. In this study, poly(d,l-lactide) (PDLLA) with high molecular weight was synthesized via ring opening polymerization, which was hot-pressed into PDLLA membrane. In order to modify the hydrophobicity of the membrane (a limiting factor for its biomedical application), an amphiphilic monomethoxyl poly(ethylene glycol)-b-poly(d,l-lactide) (PEG-PDLLA) was selected to improve its surface hydrophilicity through a simple self-assembly approach. It was found that the contact angles of the modified membrane can be well controlled by variation of PEG-PDLLA concentrations. *In vitro* cell biological study indicates that optimized cell adhesion can be achieved on the modified membrane with a contact angle of around 50° via its self-assembly with an ethanol/water solution of PEG-PDLA (35 mg ml^−1^). The surface modification of the membrane also changed its biodegradation property in the process of its incubation period up to 240 days. The surface modification method may afford an effective way for adjustment of the surface (interface) of membrane (scaffolds) of different biomaterials, beyond polylactide.

## 1. Introduction

The biological functions of cells are strongly affected by the microenvironments where they ‘live in’. Generally, cells can sense the signals of the extracellular matrix via mechanotransduction [1]. A lack of sufficient cell adhesion may block cell anchorage to the substrates, resulting in apoptosis, since it is a necessity for transferring the mechanical signals from the substrates to cells to induce actin-myosin cytoskeleton traction forces, which can be transduced into bioactive signals for adjustment of cell bioactivities [2–5]. Although cells mostly live in complex three-dimensional microenvironments *in vivo*, a two-dimensional substrate with adjustable surface state may offer a simpler and easier model to study the mediation effects on the biological activities of cells [6]. Therefore, it is important to develop a kind of biocompatible membrane to act as a two-dimensional substrate with adjustable surface properties to study the effect on mediation of cell bioactivities.

As a kind of synthetic biocompatible polymer, polylactide (PLA) is produced from 100% renewable resources. PLA can also degrade into CO_2_ and H_2_O under biological conditions [7,8]. Therefore, PLA has been approved by the US Food and Drug Administration for a wide spectrum of biomedical applications, ranging from drug delivery systems, implant material, surgical material, tissue engineering scaffolds to bone fixation devices [9–12]. However, the low wettability and surface energy of PLA is still a bottleneck to support sufficient cell attachment and proliferation, which is a main reason to hamper its successful tissue regeneration [13].

Generally, the surface modification of polymeric membranes can be achieved via physical decoration and/or chemical conjugation. For instance, different inorganic compositions [14] or organic ones [15,16] can be introduced onto a polymeric membrane through physical adsorption and precipitation. However, the decorated composition on the membrane modified by this method is not stable. In order to improve the stabilization, a polymeric membrane can be firstly pre-incubated in special solution and/or undergo surface treatment technique to introduce some functional groups, which can then be conjugated with some hydrophilic polymers [17,18]. However, the current chemical modification method is very complex, and also may damage the polymer substrates themselves. Recently, various strategies including decoration of PLA with natural materials like albumin, gelatin, collagen, or alginate have been intensely investigated to improve the biocompatibility of PLA-based biomaterials [19,20]. These methods have been found to improve cell attachment and growth [20,21]. However, the introduction of natural products onto PLA surface may introduce some complex chemical elements onto the interface, which may greatly complicate the process procedure, and also induce some immunogenic responses [6,22,23]. It is proposed that modification of PLA by a kind of synthetic polymer with known structure and composition may offer a better way to finely adjust its surface conditions to further enrich its biomedical applications.

Herein, in this study, we firstly developed poly(d,l-lactide) (PDLLA) with high molecular weight via ring opening polymerization. The resulting polymer was processed into PDLLA membrane through a hot-pressing method. After that, an amphiphilic monomethoxyl poly(ethylene glycol)-b-poly(d,l-lactide) (PEG-PDLLA) was assembled on the membrane under mild conditions (ethanol/water solution). The results indicated that, by controlling the amount of PEG-PDLLA on the PDLLA surface, the resulting modified membrane (PDLA-M) presented controllable contact angles. Cell culture evaluation indicated that PDLA-M with moderate contact angle (around 50°) enabled optimal cell adhesion and proliferation. PDLA-M membrane also displayed different biodegradability. Compared to the conventional surface modification techniques, our method is expected to have the following advantages: (i) membranes (even scaffolds) with controllable hydrophilicity can be achieved through dipping them in PEG-PDLLA solutions with different concentrations; (ii) the materials which have been introduced onto the membrane have known structure and compositions, which can be more easily approved for biomedical applications; and (iii) the modification process does not involve any toxic organic solvent. Therefore, this simple and environment-friendly approach to modification of hydrophobic membrane may not only offer a good model for investigation of its surface effect on cell functions, but also enlighten a design of novel biomaterials for tissue engineering application.

## 2. Experimental

### 2.1. Materials

d,l-Lactide and PEG-PDLLA (each block with equivalent molecular weight of 2 kDa) were purchased from Jinan Daigang Biomaterial Co. Ltd, China. Tin-2-ethylhexanoate (Sn(Oct)_2_) was obtained from Sigma-Aldrich, China. Anhydrous ether, dichloromethane and ethanol were bought from Shanghai Taitan Chem Co. Ltd, China. 3-(4,5-Dimethyl-2-thiazolyl)-2,5-diphenyl-2H-tetrazolium bromide (MTT) and 4,6-diamidino-2-phenylindole (DAPI) were ordered from Life Technology, China. C2C12 cells (mouse myoblast cell line) were obtained from American Type Culture Collection, USA.

### 2.2. Preparation and characterization of PDLA-M membrane

PDLLA was synthesized via ring opening polymerization of lactide using Sn(Oct)_2_ as catalyst at a reaction temperature of 140°C for 6 h [24]. The obtained mixture was purified through dissolving/precipitating treatment using dichloromethane and ether, followed by drying under vacuum at 60°C for 48 h to get PDLLA sample. The PDLLA was hot-pressed on a press vulcanizer (BL-6170, Bolon Instruments, China) at 180°C and 10 MPa to obtain PDLLA membrane with 0.5 mm thickness for further study.

**Scheme 1.**
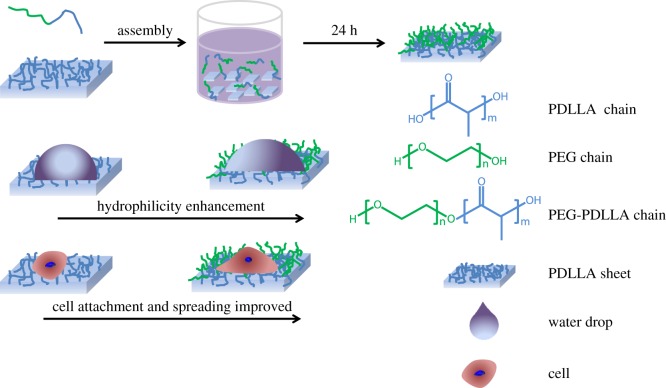
A schematic representation of how to adjust the hydrophobicity of PDLLA membrane via self-assembly with PEG-PDLLA amphiphilic copolymer for mediation of cell functions.

For preparation of the modified membrane (scheme 1), PEG-PDLLA was dissolved in a mixture of ethanol/water with a 1 : 1 volume ratio. PDLLA sheet was cut into 1 × 1 cm quadrate specimens, which were soaked in the mixture solution of PEG-PDLLA with different concentrations (0, 5, 15, 35, 50 mg ml^−1^) for self-assembly for 24 h. After that, the samples were taken out and dried in an oven at 50°C for 6 h to get the modified membranes designated as PDLA-M_0, PDLA-M_5, PDLA-M_15, PDLA-M_35, PDLA-M_50, respectively.

The molecular weight and polydispersity index of the polymers were investigated via gel-permeation chromatography with a Shimadzu Prominence HPLC instrument (Kyoto, Japan) using tetrahydrofuran as eluent with a flow rate of 1.0 ml min^−1^. Polystyrene standards were used for calibration for calculation of the weight average weight (*M*_w_) and number average molecular weight (*M*_n_).

The chemical structure of the samples was studied by nuclear magnetic resonance (NMR) spectroscopy with a NMR instrument (Bruker AVANCE III 600, Bruker Corporation, Switzerland).

The thermal analysis of the samples was conducted with a differential scanning calorimeter (DSC2910, TA Instruments, USA) at a heating rate of 10°C min^−1^. The curves were scanned from room temperature to 230°C at a heating rate of 10°C min^−1^, followed by maintenance of 230°C for 3 min, and then cooled down at the same scanning rate to −60°C. After maintaining the samples at −60°C for 5 min, the final curves of the samples were obtained by increasing the temperature to 230°C at a heating rate of 10°C min^−1^.

The hydrophilicity of the self-assembled membrane was evaluated by measurement of the static contact angle with a contact angle meter (JC2000D2, Zhongchen Instruments, China). Images of the water droplets on the membrane were recorded, and the results were processed by the image acquisition system.

Sample sheets of 250 mg were incubated in 10 ml phosphate buffer solution (PBS, pH = 7.4) in a heating incubator (37°C) at shaking rate of 80 rpm. At a specific interval, the samples were washed with ultrapure water and lyophilized until constant weight. The weight of specimens and the pH values of the degradation media were recorded. At each measurement, incubation medium was renewed with 1 ml fresh PBS. Sample degradation was evaluated by the weight loss ratio of the samples calculated according to the following equation:

2.1 $$\text{weight loss}\;(\%)=\frac{w_{0}-w_{t}}{w_{0}}\times 100\%,$$

where *w*_0_ is the weight of the original samples used for incubation, *w_t_* is the sample weight at the incubation time *t*. Five parallel specimens were conducted for each group.

### 2.3. Cell biological study

C2C12 cells were cultivated in Dulbecco's modified Eagle's medium supplemented with 10% fetal bovine serum, 100 U ml^−1^ penicillin, 100 mg ml^−1^ streptomycin at 37°C in a humidified atmosphere and 5% CO_2_. The samples were sterilized by ^60^Co (cobalt ion with a mass number of 60) radiation at 10 kGy before biological analysis.

Cell viability and proliferation on the sheets modified was evaluated via the MTT assay. Briefly, the cells were seeded on the polymer membrane at a density of 1.0 × 10^4^ cells per well in a 24-well cell plate. After 24 h incubation, 100 µl MTT (5 mg ml^−1^) was added into each well and was further incubated for 4 h. After that, cell medium was removed and replaced with 100 µl dimethyl sulfoxide to dissolve violet formazan crystals at 37°C in oscillation container. After standing for 10 min at room temperature, the plate was measured at 492 nm by an enzyme-linked immunoadsorbent assay plate reader (SPECTRAmax 384, Molecular Devices, USA). Results were presented as the percentage ratio of OD_sample_/OD_control_ × 100% (*n* = 3).

To investigate the cell morphology, cells were seeded on the sample membranes at a density of 5.0 × 10^4^ cells per well in a 24-well plate. After 24 h incubation, cell media were removed from the wells, washed with PBS thrice and fixed with glutaraldehyde solution (2.5% glutaraldehyde). Scanning electron microscopy (SEM, S-4800, Hitachi, Japan) was employed to observe cell morphology. For fluorescent imaging of the cell morphology and spreading, the fixed cells were treated with FITC-phalloidin (2 µg ml^−1^, Sigma–Aldrich, USA) to stain cell cytoskeleton for 45 min at 37°C, followed by washing with PBS thrice. Cell nuclei were stained with DAPI (5 µg ml^−1^) for 10 min at room temperature and washed with PBS five times. The fluorescent images of the cells were obtained using a confocal laser scanning microscope (Nikon A1R, Japan).

### 2.4. Statistical analysis

The significant differences of the results were analysed via one-way analysis of variance (ANOVA).

## 3. Results and discussion

### 3.1. Preparation and characterization of PDLLA-M membrane

**Figure 1.**
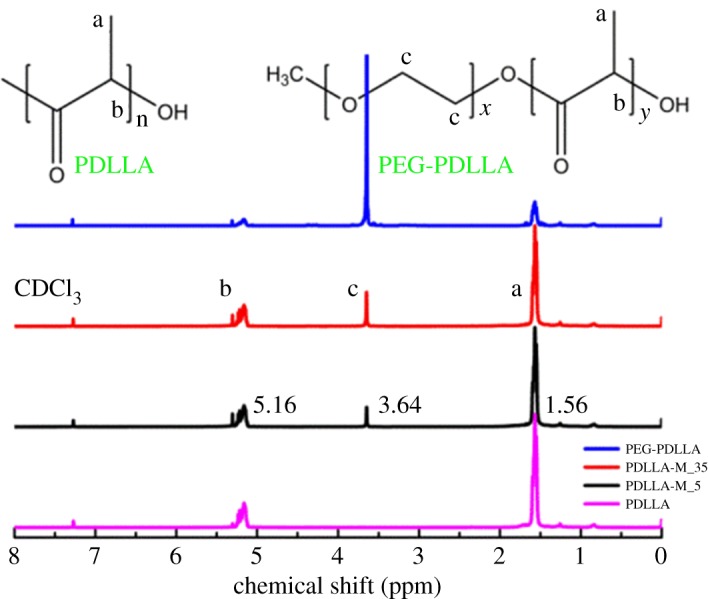
^1^H NMR spectra of PDLLA, PEG-PDLLA, PDLLA-M_5 and PDLLA-M_35 membrane.

The chemical structure of PDLLA was firstly characterized by NMR analysis. As can be seen from figure 1, PDLLA sample gave the peaks at 1.56 and 5.16 ppm which corresponded to methyl and methane protons, suggesting the successful synthesis of PDLLA [25,26]. Gel permeation chromatography analysis indicated that PDLLA had a high number average molecular weight of 201 kDa with a polydispersity index of 2.12 (table 1).

**Table 1.** The number-average molecular weight (*M*_n_), weight-average molecular weight (*M*_w_) and polydispersity index (PDI) of PDLLA.

sample identity	*M*_n_ (kDa)	*M*_w_ (kDa)	PDI
PDLLA	201	426	2.12

Although the good biodegradability and biocompatibility of PLA endow it with broad biomedical applications, its strong hydrophobicity results in poor cellular affinity and cell recognition ability, which is a bottleneck for further applications [27]. A simple method was used in this work to adjust the hydrophilicity of PDLLA via self-assembly of its block copolymer with PEG (PEG-PDLLA) onto the PDLLA membrane in ethanol/water solution. As shown in figure 1, the modified membrane (PDLLA-M) clearly presented a characteristic peak of PEG at *δ* = 3.64 [25,28], suggesting that PEG-PDLLA has been assembled onto the PDLLA membrane. The increase of PEG-PDLLA amount for self-assembly enhanced the relative intensity ratio of PEG to PDLLA, indicating the effectiveness in self-assembling the block copolymer onto PDLLA membrane via this approach.

**Figure 2.**
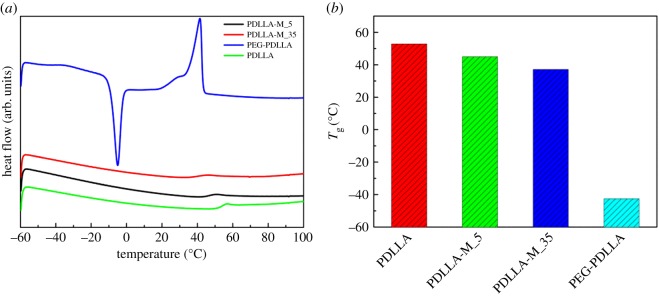
DSC thermograms of neat PDLLA and PDLLA-M membranes decorated with different amounts of PEG-PDLLA (*a*), and their effects on glass transition temperatures (*T*_g_) of the modified membranes (*b*).

To get an insight into the thermal properties and miscibility of PDLLA and PEG-PDLLA in the membranes, their crystallization and melting temperatures were investigated by DSC analysis. As shown in figure 2, PEG-PDLLA displayed a glass transition temperature (*T*_g_) at around −45°C, a crystallization temperature at −5°C, and a melting temperature at around 40°C. After self-assembly, all these peaks of pure PEG-PDLLA in the modified membrane disappeared, suggesting that PEG-PDLLA has been well dispersed on the PDLLA surface which may disorder its crystalline structure. The increase of the amount of PEG-PDLLA led to the gradual decrease in the glass transition temperatures, indicating PEG-PDLLA and PDLLA had good miscibility [29–32]. This may be the main reason why PEG-PDLLA can act as an effective modifier for adjustment of the hydrophilicity of hydrophobic PDLLA membrane.

**Figure 3.**
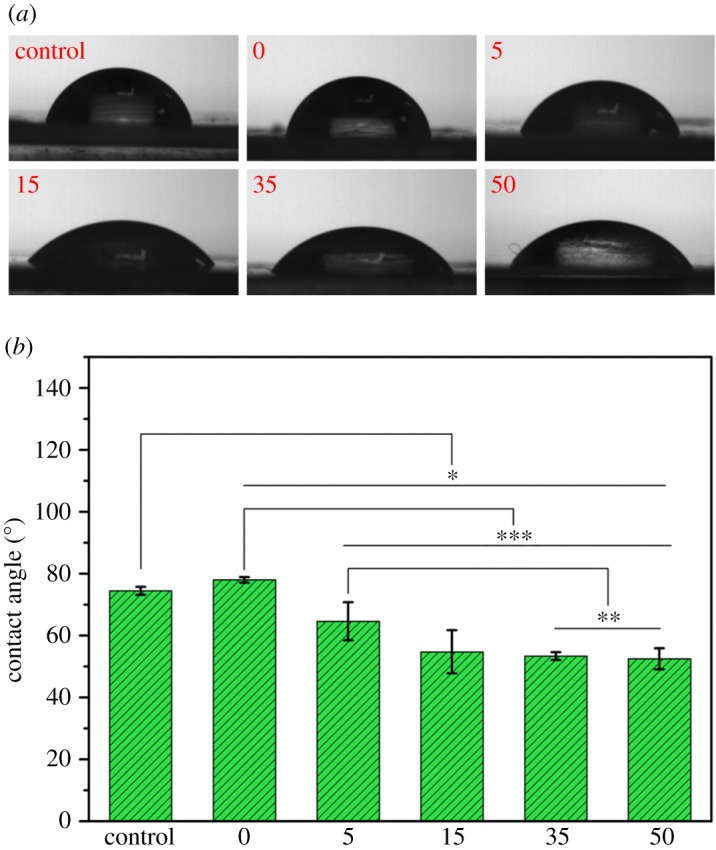
Contact angles of the neat PDLLA and PDLLA-M membranes modified with different amounts of PEG-PDLLA (mean ± standard deviation (*n* = 4), *p < 0.03, **p < 0.02, ***p < 0.002).

It is known that the surface/interface conditions of materials play an important role in mediation of cell behaviours [33]. In order to check if this kind of modification can afford membranes with adjustable hydrophilicity, the contact angles of the membranes were studied via variation of PEG-PDLLA amount for membrane decoration. The increase of PEG-PDLLA concentrations from 0 to 35 mg ml^−1^ can effectively decrease the contact angles of the membranes from 74.5 ± 2.4 to 50.0 ± 2.3° (figure 3). As such, the introduction of the block PEG-PDLLA on the PDLLA membrane allowed for the controllability of its hydrophilicity, which may be useful for fabrication of new biomaterials to guide cell behaviours to achieve better bioactivities for tissue engineering applications. It has been reported that substrate with contact angle 40–60° is beneficial to cell affinity probably because cell membrane which mainly consists of phospholipids and cholesterol may assume moderate hydrophilicity [33,34].

### 3.2. Cell biological performances of PDLLA-M membrane

**Figure 4.**
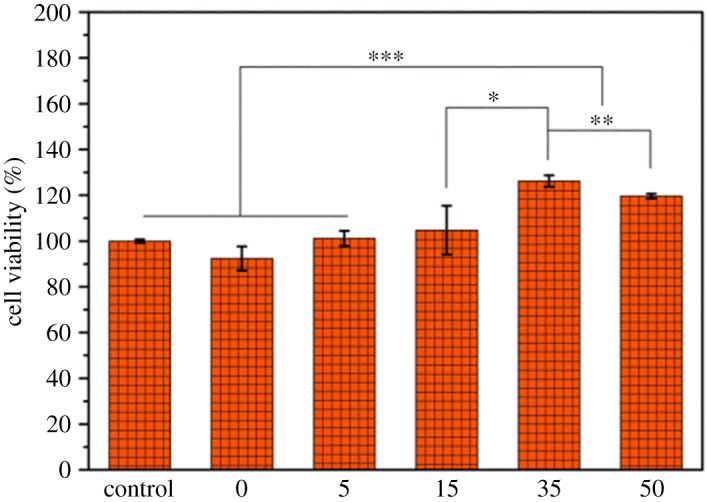
Cell viability of C2C12 cells after 24 h incubation on neat PDLLA and PDLLA-M membranes via MTT assay (mean ± standard deviation (*n* = 3), *p < 0.03, **p < 0.02, ***p < 0.002).

For biomedical application, materials should present high biocompatibility [35,36]. Therefore, the cytotoxicity of neat PDLLA and PDLLA-M membranes was quantitatively evaluated through the MTT assay using C2C12 cells as model cells. As shown in figure 4, after 24 h incubation, C2C12 cells on all the membranes maintained a high cell viability (above 100%), suggesting their high cytocompatibility. The PEG-PDLLA-treated membranes gave higher cell viability than the pure PDLLA membrane, probably due to their improved hydrophilicity which may benefit cell adhesion and proliferation [37–39]. The optimal cell viability occurred on the PDLLA-M membrane when it was decorated with 35 mg ml^−1^ PEG-PDLLA. This is because excessive PEG amount on the substrate may reduce protein adsorption capacity, hampering the affinity to cells [40,41].

**Figure 5.**
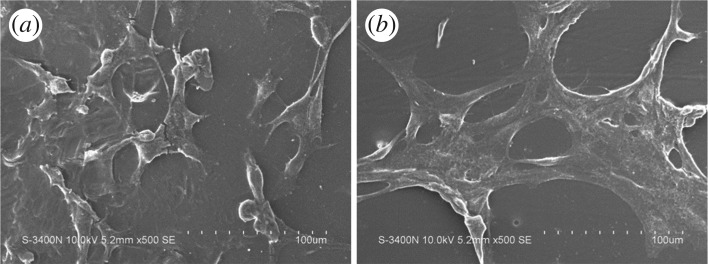
SEM micrographs of C2C12 cells on (*a*) neat PDLLA and (*b*) PDLLA-M_ 35 membranes after 24 h incubation.

**Figure 6.**
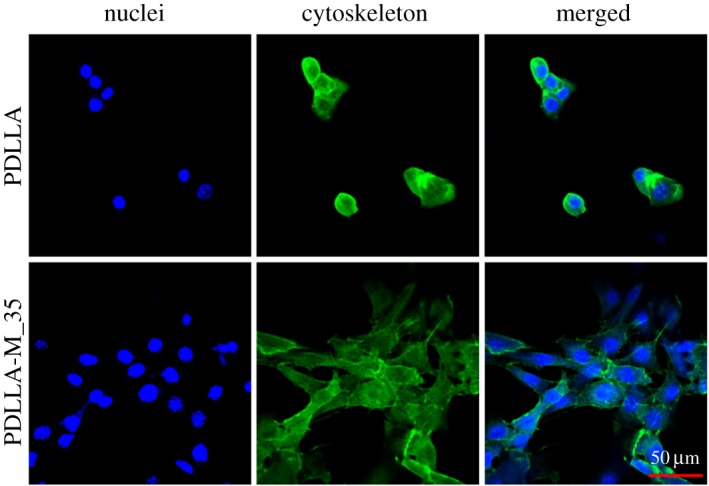
Fluorescent images of C2C12 cells cultured on neat PDLLA and PDLLA-M_35 membranes after 24 h incubation.

Cell spreading is a key factor for regulation of cell functions (e.g. stemness maintenance or stem cell differentiation, cell proliferation or apoptosis) during tissue/organ development [42]. Besides their stiffness [43] and/or topography [6], the hydrophilicity of substrates has an important effect on mediation of cell behaviours. Therefore, the cell spreading and the morphology of C2C12 cells cultured on neat PDLLA and PDLLA-M_35 membranes were investigated by SEM observation. As shown in figure 5, cells cultured on the PDLLA substrate adopted a relatively round shape with relatively poor cell adhesion, while cells on the PDLLA-M_35 membrane presented an elongated morphology, suggesting cells can achieve good spreading, which may benefit cell growth. The fluorescent images of cells clearly indicated that few cells with round shape were found on the PDLLA membrane, while various cells with well spreading morphology can be seen on the PDLLA-M_35 membrane (figure 6). Cell biological results again proved that the way of surface modification of PDLLA substrate by PEG-PDLLA assembly is very effective not only in adjustment of its surface hydrophilicity but also in mediation of the following cell bioactivities, which may be useful to extend its biomedical applications.

**Figure 7.**
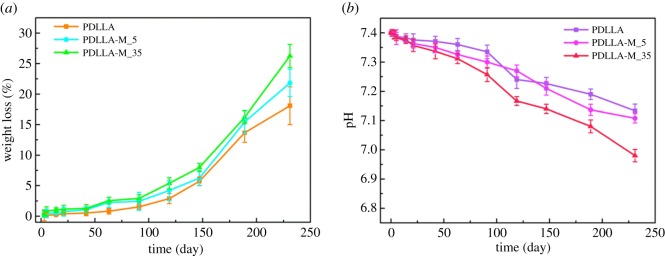
(*a*) Weight loss and (*b*) pH value change of neat PDLLA and PDLLA-M membranes during their incubation in PBS of pH 7.4 at different incubation periods.

Since the degradability of materials is very important for their biomedical applications, the biodegradation process of the pure PDLLA and modified membranes was studied by investigation of their weight loss as a function of incubation time in PBS (pH 7.4). It can be seen from figure 7 that all the membranes displayed a gradual weight loss until 240 days. The increase of PEG-PDLLA modifier amount seemed to accelerate the degradability of PDLLA membrane, which may be associated with the enhanced hydrophilicity increasing the water adsorption ability and accelerating the erosion process of the membrane. Interestingly, the membranes did not lead to a drastic pH decrease during the incubation process, which may reduce the inflammation response often occurring in the corresponding PLLA materials [44,45], probably because their heterogeneous morphology and uncontrollable biodegradability can cause a burst of lactic acid accumulation.

## 4. Conclusion

In summary, we have developed a simple and effective approach to adjust the hydrophilicity of PDLLA by self-assembling amphiphilic PEG-PDLLA onto membranes of PDLLA. Variation of PEG-PDLLA decoration amount from 0 to 50 mg ml^−1^ allows for adjustment of the membrane contact angle from 74.5 to 50.0°. Results indicated that the membrane which was modified with 35 mg ml^−1^ of PEG-PDLA offered an optimal cell adhesion, spreading and proliferation, where its contact angle is about 50.0°. The surface modification method can also affect the biodegradation process of the PDLLA membrane. The effectiveness in combinative improvement of the surface condition as well as degradation property of PDLLA may offer a good way for fabrication of biodegradable membranes (scaffolds) with controllable surface (interface) for tissue engineering applications.

## Supplementary Material

Raw Data

## Data accessibility

The datasets supporting this article have been uploaded as part of the electronic supplementary material.
